# Materia Medica and Therapeutics—Medical Chemistry—Toxicology and Legal Medicine

**Published:** 1861-05

**Authors:** Emil Fischer


### MATERIA MEDICA AND THERAPEUTICS; MEDICAL CHEMISTRY; TOXICOLOGY AND LEGAL MEDICINE.

BY EMIL FISCHER, M.D.

### MATERIA MEDICA AND THERAPEUTICS.

I.—*On Anacahuite Wood.* By JNO. M. MAISCH. (American Journal of Pharmacy, March, 1861.)

*Note on Anacahuite Wood, a Reputed Remedy for Consumption.* By DANIEL HANBURY, F.L.S. (London Pharmaceutical Journal, February, 1861.)

1. "Some time in August or September last, the New York *Criminal Zeitung* published a correspondence from Berlin, Germany, which contained a statement to the effect that the Prussian consul at Tampico, Mexico, had notified his government of a wood, which is called there anacahuite, and is extensively employed with the most beneficial results in tubercular consumption. This report was considered by the government of Prussia of sufficient importance to determine on testing its efficacy in the Charité, the celebrated hospital in Berlin. The wood, it is asserted, is rare in Mexico, and the agents of Prussia have seized upon it to such an extent that it is now difficult for others to obtain.

"A subsequent correspondence of the above-mentioned paper, dated Berlin, October 5th, 1860, stated that the experiments with the anacahuite wood had, as yet, not led to a satisfactory result, but that they were to be continued, because it was hardly to be expected that the consul should have made such positive assertions without being satisfied of their correctness; it was added that guaiac wood had, by some druggists in Germany, been fraudulently sold instead of anacahuite.

"The *Criminal Zeitung*, of February 1st publishes a paper by Dr. Krog, of New York, formerly of Berlin, which appears to be of so much interest as to warrant its translation for the American Journal of Pharmacy.

"'During the summer of the past year, when the above wood was first brought to Europe, the writer has had the opportunity to observe its effects in the Berlin Charité, and to satisfy himself of the unusually favorable results. The first experiments did not prove to be so effectual as was expected, and it was supposed that a part, at least, of the alleged excellent success in Mexico must be ascribed to its climate; but subsequently it became evident that this difference was to be accounted for by the mode of preparing the wood. A simple infusion is not sufficient to extract the active principles; and heating to boiling will not entirely accomplish the object, which is attained only by continuing the boiling for a quarter or half an hour. Such a decoction has proved very efficacious; it produced the complete resorption of the tubercles in the first stage of consumption, and afforded great relief of their distressing condition to those further advanced. These facts suffice to make it desirable that this remedy might be extensively employed here for the benefit of the numerous patients of this class.'

"Such information, coming from a medical man who had witnessed the experiments, is entitled to consideration, and in view of the importance of the subject, I asked for further information, and received the following letter, which speaks for itself.

"'New York, Feb. 5th, 1861.

"'Dear Sir:—In reply to your favor, I hasten to inform you that the anacahuite wood certainly merits the attention which it has lately received. My experience chiefly extends to its medicinal effects. At present I am unable to give the name of the mother-plant; to judge, however, from some pieces of genuine wood now before me, I am inclined to feel justified in placing it in the natural order of Papilionaceæ.

"'It is believed in Berlin that gratifying results have been obtained with this remedy in the first stages of phthisis pulmonalis. It is given in the form of decoction, namely, ʒvi to ℥j of the wood to 12 to 14 oz. of water, boiled down to ℥v, and this is taken 2 to 4 times daily, according to circumstances, combined with other remedies. It is requisite to continue the use of this remedy for several months, and to observe a diet in accordance with the nature of the disease.

"'Two weeks ago I wrote to Berlin for the purpose of learning the latest observations with this wood, and I am ready, with pleasure, to communicate to you the results on their arrival.

"'Yours, etc.,

"'KROG, M.D., 337 Tenth St.'"

It is proper, however, to state that Professor Bock, of Leipzig, opposes the use of anacahuite, insisting that in pulmonary consumption relief can *only* be found in the strictest regimen, by partaking of suitable food, breathing a pure and warm atmosphere, using moderate exercise, and attending scrupulously to a mental, intellectual, corporeal, and sexual rest.

2. Mr. Daniel Hanbury, after referring to the recent introduction of this new drug into the market, and to the great demand for it in Germany, describes it as follows:—

"Anacahuite wood is a production of Tampico, whence, in fact, all the supplies that have reached Europe have been shipped. Its botanical origin is at present unknown. Dr. Otto Berg, who has elaborately described its external characters and anatomical structure, (*Bonplandia*, 15th Oct., 1860, p. 302,) thinks that from the organization of the bark and wood it is probably derived from some papilionaceous tree, although there are only general appearances that guide him to such an opinion. It is to be hoped, however, that this question may be soon set at rest by good, dried specimens of the flower, fruit, and leaf of the tree being obtained from Tampico, and submitted to some competent botanical authority in Europe. Anacahuite wood, as I have seen it, consists of truncheons of about two feet long, varying from the thickness of a finger to that of a man's arm. The wood is covered with a thick, fibrous, grayish-brown bark, coarsely furrowed longitudinally with deep cracks, and so tough that it may be stripped off in pieces of considerable length. A white, pulverulent matter, resembling an efflorescence, occurs between the layers of liber, from which it escapes as dust when the bark is torn. When one examines a transverse section of a truncheon, one perceives the bark to be of considerable thickness and to consist of two more or less defined zones—the inner more compact. The wood is of a pale brown, marked with concentric zones, which, however, are too little distinguished from one another to be counted with any certainty. The pith is frequently eccentric; its transverse section sometimes shows a stellate form.

"Anacahuite wood is inodorous and insipid. A strong decoction is transparent and of a sherry-brown color; it is blackened by a persalt of iron, but neither a solution of gelatin nor of iodine affects it. The taste of the decoction is extremely slight and unremarkable, so that one may reasonably be permitted to doubt the extravagant, though, if true, very gratifying assertions regarding the virtues of the drug. The experiments, indeed, that have been instituted in the great hospital at Berlin have hitherto had, as I am informed by Dr. C. G. Mitscherlich, no satisfactory results. The details of the cases have not, however, yet been published."

II.—*Nitric Acid in Intermittent Fever.* By WILLIAM A. HAMMOND, M.D., Professor of Anatomy and Physiology in the University of Maryland.

In an article, contained in the *Maryland and Virginia Medical Journal* for February, 1861, Professor Hammond recommends the use of nitric acid in intermittent fever. He publishes a table showing the results obtained in the treatment of a number of cases of this disease, and accompanies it with the following remarks:—

"The table forms the basis of a report made about four years since to the Surgeon-General of the Army, and has never been published. The cases were treated at Fort Riley, Kansas Territory, in the post hospital, then under my charge, in a period of six weeks in summer.

"Upon referring to the table, it will be seen that in all forty-one cases were treated, ten of these being of the quotidian type, and thirty-one of the tertian. Thirty-two cases were treated with the nitric acid, and nine with the sulphate of quinine. Of the cases cured by nitric acid three had previously used quinine without effect, and of those in which quinine had proved successful nitric acid had been employed without benefit in two, and in one other had to be omitted on account of causing nausea, heart-burn, etc.

"The average period of treatment before the disease was permanently arrested was the same with each remedy—three days. The nitric acid was uniformly given in doses of ten drops (properly diluted with water) three times per day, the quinine in doses of eight grains three times per day.

"Besides the fact that the nitric acid was equally successful with quinine in arresting the disease, the difference in the cost of the two articles is so greatly in favor of the former substance as to render it an object of importance to make its curative properties more widely known.

"Nitric acid was first used as an anti-periodic by Dr. E. S. Bailey, of Indiana. Its peculiar properties were brought to the notice of the profession by Dr. George Mendenhall, in the *Western Lancet* for August, 1854. A notice of the discovery is also contained in the *American Journal of Medical Sciences* for October, 1854." * * * * * * * * * * * *

"Since the foregoing cases were treated, I have very frequently employed nitric acid in the treatment of intermittent fever, and have rarely been disappointed in my expectations of its curative action. In fact, in simple uncomplicated intermittent, I seldom have occasion to use anything else.

"In cases of enlargement of the spleen, consequent upon frequent attacks of the ague, the remedy in question has, in my hands, proved very advantageous.

"The whole subject is one of great importance to the physicians of malarious districts, and I trust will sufficiently engage their attention as to induce them to test the curative power of nitric acid in those cases of intermittent fever which may fall under their charge, and to add to the sum of our knowledge by reporting for the information of others the results at which they may arrive."—(*Maryland and Virginia Medical Journal*, February, 1861.)

### III.—*Kukui, or Kekune Oil.* By M. C. Cooke.

Now that the oil of Aleurites triloba is spoken of so highly in France as a purgative oil, a few particulars concerning it may not prove uninteresting.

The plant producing the fruits from whence this oil is extracted belongs to the natural order of Euphorbiaceæ, and is plentiful in the Sandwich, Society, and other groups of islands in the Southern Seas. It is also to be met with in some parts of Jamaica and the East Indies. The oil has been for some time known in Jamaica as Spanish walnut oil, and in India as Belgaum walnut oil. In Ceylon the oil is called Kekune oil, and in the Sandwich Islands Kukui oil. The tree is known in some parts of Polynesia as the candle-nut tree. The fruits are nearly as large as a walnut, and the kernel is inclosed in a thick, hard shell. These nuts are often strung together by the natives, and burnt, without any other preparation, as torches. In the history of the mutiny of the Bounty, it is stated that the rooms in Pitcairn's Island were lighted up by torches made of "doodoe" nuts, strung upon the fibres of the palm leaf, forming a good substitute for candles. These nuts are also so strung and used by the San Blas Indians in Central America, and a child is in attendance to knock off each nut as it becomes burnt out.

The following is the method adopted in obtaining the oil in Jamaica. Each nut is carefully cracked or broken, and the kernel as carefully separated from the hard shell, lest the latter, having a brown dye quality, should affect the color of the oil. The kernel is then put into a large mortar and pounded as fine as possible. It is afterward thrown into a caldron with plenty of water, and boiled. It is allowed to simmer for hours, until all the oil is well extracted and floats on the surface. Meanwhile, and until all is gathered together, the oil is skimmed off into another clean vessel. The oil thus collected is then boiled over again in a smaller vessel for a short time, in order to throw off any aqueous particles remaining after the first skimming. If the oil is not then perfectly pellucid, it is run through blotting-paper. Eight quarts of kernels will yield about three pints of oil. The yearly produce of oil in the Sandwich Islands is about 10,000 gallons. It has been shipped to the markets of Chili, New South Wales, and London, but hitherto without much profit. It realized about £20 per imperial ton in London. In 1843 about 8620 gallons were shipped from Honolulu, valued at 1*s.* 8*d.* per gallon.

This oil has been used as an artist's oil, for which purpose it is said to possess valuable qualities, although it cannot be applied as a drying oil It is only lately that attention has been called to its medicinal properties. It is purely purging, and, not like the croton, jatropha, caper-spurge, sandbox and other euphorbiaceous oils, productive of vomiting at the same time. It is affirmed to be as mild as castor oil, and being more fluid, is better to take; it is without either taste or smell.

The nuts have, within the past twelve months, been sold in the London market under the name of kukui nuts, and there is no doubt that, upon inquiry, some of the oil could be procured, and it evidently well merits the attention of the profession. A purgative oil which shall possess all the advantages, and none of the disadvantages of castor oil, is a desideratum worthy of being secured.—(*London Medical Review*, and *Pharm. Journ.*, November, 1860.)

### IV.—*Steadine a Substitute for Hog's Lard.*

Steadine is but a contraction of the word stearaïdine, resembling fat, and is prepared as follows:—

| | |
|---|---|
| Lard . . . . . . . . . . | 3½ ounces. |
| Water . . . . . . . . . . | 3½ " |
| Soda deprived of its carbonic acid by lime . . | 15 grains. |

The soda is weighed and used dry. It should be melted in about four drachms of water; the lard is then gradually added alternately with the remaining water. The operation of this mixture is both swift and simple; in ten minutes four pounds of steadine may be prepared. This new adipose substance presents the appearance of a whitish, fatty compound, inodorous, insipid, and intermediate between cerate and lard. Its consistency, soft at first, soon acquires more firmness; it is not, like axunge, liable to liquefy during warm and to harden in cold weather. It indefinitely preserves its color and density, unless left constantly exposed to the atmosphere—a practice injurious to other pomades and fatty matters, which turn rancid from prolonged contact with the oxygen of the air.

The slight addition of the alkaline ingredient is undiscoverable from taste or from examination with test-paper, being entirely saturated by the fatty excipient. It suffices, however, to create a new fatty substance of a mixed nature, a medium between fats and bodies soluble in water. A new and solid species of glycerin is thus produced, which is to a certain extent soluble in oils and water. This double property renders it capable of being in the preparation and use of pomades as serviceable as glycerin itself for oils and liniments. Ointments containing metallic bases, oxides, chlorides, sulphurets, iodides, salts, etc. remain unaltered; the iodide of potassium pomade preserves its whiteness, and does not lose its iodine. For the purpose of manipulation, it will be found most convenient. Insoluble powders mix with it promptly and with great accuracy. It is more readily combined with vegetable powders than lard, a bad solvent of their active principles. Soluble salts, extracts which require previous dilution in water, can be immediately and completely associated with steadine, whereas, when hog's lard is used, the repulsion between the ingredients is so great that a very protracted and persevering manipulation is necessary before even a badly-assorted union can be effected.—(*Journal of Practical Medicine and Surgery*, and *Pharmaceutical Journal*, London, December, 1860.)

V.—*Tincture of Iodine in Acute Superficial Phlebitis.*

The Russian medical journals have lately published several cases illustrative of the good results of the external employment of tincture of iodine in acute superficial phlebitis. One of the examples cited is of sufficient practical importance to merit translation: "A peasant, of robust frame, and of about thirty years of age, was attacked with pleurisy, and in consequence bled in the arm. During the evening succeeding the venesection, pain, redness, and swelling appeared, both in the neighborhood of the wound and over the surface of the limb. Five days after the bleeding, as these symptoms had progressively increased in gravity, resisting treatment, the patient was admitted into the Hospital Marie, at St. Petersburg. Dr. Spoiret, author of the collection of cases, and chief medical officer of this establishment, found the whole arm enormously swollen, the skin tense and dark red in color; in the situation of the vein implicated was seen a broad line, of a livid, violet hue, to the touch feeling hard, cord like, and pressure occasioning great pain. The swollen and discolored track could be followed to the armpit, evidencing an inflammatory condition of the brachial vein. Violent fever was likewise present, with a hard, wiry pulse, headache, thirst, and constipation. A treatment of leeching, mercurial unction, fomentations, purgation, and muriatic-acid mixture, pursued for four days, proving utterly ineffectual, the fever having increased in gravity, and symptoms of purulent infection setting in, a total change was ordered in the *régime;* and while sedatives were administered internally, applications of tincture of iodine, renewed every three hours, were freely made along the whole course of the inflamed vein. At the end of two days the inflammation had considerably abated; a kindly suppuration commenced, and the case did well." Although bleeding, like dueling and such other relics of barbarism, is daily becoming less and less common in England, the one yielding to the advance of science, and the other to the march of civilization, and although the term "leech" is becoming daily less and less true in its application to members of our profession, still cases of phlebitis from superficial injuries in unhealthy subjects are far from being rare, and the above citation, from the *Russian Medical Zeitung*, I deem quite worthy the attention of your readers.—(*Lancet*, December 22, 1860.)

### VI.—*Thapsia Plaster.*

This is a new therapeutical agent, the active principle of which is derived from Thapsia garganica, an Algerian plant. It is a most energetic revulsive, the effects of which can be graduated by the duration of its application, replacing blisters in serious affections, and rubefacients in the milder ones. It induces an erythema, which is speedily followed by an abundant and salutary miliary eruption. Its action is rapid, more certain than that of croton oil, and its employment is exempt from the numerous inconveniences attributed to some other external agents. It may be employed in any cases in which revulsion is indicated, but it is especially useful in diseases of the chest, rheumatism, and arthritis; it is also serviceable in the affections of children.—(*Med. Times and Gaz.*, November 17, from *Gazette des Hôp.*, No. 60.)

---

### MEDICAL CHEMISTRY.

I.—*On the Detection of Sugar in Urine.* By MR. ATTFIELD. (London Pharmaceutical Journal, January 1, 1861.)

*On the same.* By DR. BENCE JONES. (London Pharmaceutical Journal, February 1, 1861.)

*New Test for Diabetes.* By DR. E. C. BIDWELL. (Boston Medical and Surgical Journal, Nov. 22, 1860, and New Orleans Med. News and Hosp. Gazette, Jan., 1861.)

1. The *London Pharmaceutical Journal* for January notices a communication from Mr. Attfield, which has also appeared in the *Chemical News*, having reference to Boettger's test for sugar in urine. It will be remembered that his test consists in adding a few grains of ordinary trisnitrate of bismuth to a portion of the urine, then pouring in an equal volume of a strong solution of carbonate of soda, and heating the mixture. The presence of sugar is indicated by the formation of a deep brown or black color, due to a reduction of the oxide of bismuth. Boettger originally devised this process to obviate some of the sources of error which frequently attend the application of Trommer's test. It is well known that the presence of certain constituents of the urine, and especially of uric acid in abnormal quantity, may cause a reduction of the oxide of copper in the application of Trommer's test, without sugar being really present. Oxide of bismuth was found to be certainly not affected by uric acid, and to that extent therefore might be considered as preferable to oxide of copper. Mr. Attfield has been engaged in some experiments on the applicability of Boettger's test. He states that it is far more delicate than Trommer's. He finds that by it abundant indications of the presence of sugar can be obtained in an aqueous solution so dilute as to give no reaction with Trommer's test. But he also finds that the presence of albumen in the urine will cause a reduction of the oxide of bismuth, and introduces therefore a source of error. He states that the action of albumen is far less energetic and complete than that of sugar, but that nevertheless blackening does occur, and to a greater extent than could be accounted for by the influence of the sulphur present in the albumen. Moreover, some of the constituents of healthy urine appear to interfere to a certain extent, for the author states that "nearly all specimens of urine color more or less the basic nitrate of bismuth." Mr. Attfield therefore objects to this test as a means of detecting sugar in urine, because of its extreme delicacy, and because other substances, and especially albumen, may produce the same reaction. For the mere detection of sugar in alkaline solutions generally, it is however exceedingly sensitive. The author concludes his communication by recommending Trommer's test, which he considers "for medical purposes fulfills all desirable conditions."

There can be no doubt, however, that Trommer's test is subject to certain objections. Mr. Attfield, in his paper, does not mention the test commonly known as Moore's, which consists in adding to the urine an equal volume of liquor potassæ, and boiling for some minutes, when, if sugar is present, a peculiar and very characteristic brown coloration is produced. This test is subject to many less objections than either of the above, and is now very commonly employed in the examination of urine, and for the detection of grape sugar generally. This test possesses, in addition to its being very trustworthy, one great advantage, namely, that it can be almost as readily used for determining the amount of sugar as for recognizing its presence. This depends upon the fact, that the depth of color produced bears a strict relation to the amount of sugar present. If, therefore, a standard solution of grape sugar is prepared, and a given quantity colored to the fullest extent by boiling with potash, upon taking an equal quantity of the urine and treating it in a similar manner, by comparing the colors, and diluting until the tint is equal, the proportion of sugar present in the urine is readily arrived at.

2. At a recent meeting of the Chemical Society, a paper was read by Dr. Bence Jones, on the presence of sugar in the urine. The author described a number of experiments which he had undertaken with a view, first, of ascertaining the most delicate process for the detection of minute traces of sugar when added to urine; and, secondly, for obtaining further proof of the correctness of Brücke's statement, that sugar is always present, and is a normal constituent of healthy urine. His principal conclusions were as follows: Lehmann's process for detecting sugar in the urine by extracting the evaporated residue with absolute alcohol, and precipitating the sugar therefrom in the form of potash sugar, by means of alcoholic potash, cannot be employed when small quantities are present in large quantities of urine. The process of fermentation is stopped by the residue of the urine, by much urea, and still more decidedly by oxalate of urea. Half a grain of sugar in water can be detected by the alcohol produced, and may be estimated by the carbonic acid produced, but much larger quantities may be entirely overlooked in concentrated urine. In decolorizing urine for examination in the polarizing saccharometer some sugar is always lost. Animal charcoal removes sugar in proportion to the amount of charcoal used. This sugar may be recovered by washing with boiling water. Two-thirds of the sugar in urine may be lost by Robiquet's method of decolorizing with basic acetate of lead and ammonia. Pettenkofer's test for sugar, by means of cholic or glycocholic acid and sulphuric acid, is the most delicate known. Two-thirds of a milligramme may be detected in a little distilled water, and the presence of a small amount of urinary coloring matter does not affect the reaction. Trommer's test with sulphate of copper and potash is capable of discovering one-twentieth of a per cent. of sugar in urine, but when very small quantities of sugar are in solution with muriate of ammonia or urea, the reduction of the oxide is not perceived. Brücke's alcohol process was not found to be satisfactory, but his lead process furnished excellent results. The urine is precipitated first with neutral acetate of lead, then with basic acetate of lead, and lastly with ammonia. The ammoniacal precipitate contains the sugar, which is extracted by treating the precipitate with oxalic acid, or preferably by sulphuretted hydrogen. By Brücke's process one-seventh of a grain of sugar added to 200 cubic centimetres of urine could be detected, and two-thirds of all the sugar added could be recovered. Moreover, the sugar is obtained free from salts, so that it can be fermented, and free from color, so that it may be examined by the saccharometer. The presence of sugar could be readily ascertained by this process in 1000 cubic centimetres of urine. The sugar separated by Brücke's process from 1000 cubic centimetres of the urine of a healthy man was estimated by the reduction test to vary from 1·4 to 2·2 grains, and in that of another man to vary from 2·3 to 3 0 grains. The sugar separated from 5000 cubic centimetres of the urine of one healthy man gave from 7 to 8 degrees of rotation in the saccharometer, and that of another healthy man from 10 to 11 degrees. The sugar extracted from 14,000 cubic centimetres of healthy urine yielded by fermentation 1·8 grains of carbonic acid, together with a recognizable quantity of alcohol. These and other experiments fully confirmed Brücke's statement as to the habitual presence of sugar in healthy urine. Hence diabetes must be regarded as an exaggeration of a healthy state, and not as a distinct and peculiar condition of the system.

3. Dr. E. C. Bidwell communicates to the Boston *Medical and Surgical Journal* (Nov. 22, 1860,) a process which he claims as a new test for glucosuria. His proposed test, he says, if not pre-eminently scientific, is nevertheless facile and reliable. He converts the saccharine element of diabetic urine into *caramel*, by heat. Upon a clean slip of tinned iron are placed one or two drops of the suspected material, which is then held over a spirit-lamp; the fluid will speedily evaporate, leaving scarcely a trace upon the surface of the metal. The application of the heat is continued, and in a few moments after the completion of the desiccation, a spot, about an inch in diameter, over which the drop has spread with the first ebullition, will gradually assume a rich, reddish-brown color, with a brilliant lustre, having the appearance of Japan lacquer. The application of a stronger heat produces a darker color, but the lustre continues until the intensity of the heat decomposes the substance. Dr. Bidwell claims great success in his experiments with this new method, which is far superior to those tests founded on either fermentation, or on the reduction of metallic oxides, which latter, besides being complicated and inconvenient for clinical use, are liable to various fallacies.

---

### TOXICOLOGY AND LEGAL MEDICINE.

I.—*On the Question of Quantities in Toxicology.* By M. LOUIS ORFILA.

When by chemical analysis the expert has determined the presence of poison either in the cadaver or in the matters vomited, he is required to declare whether the quantity of poison found or the quantity swallowed is sufficient to cause death or produce the symptoms observed. In order to establish the sense in which the expert should reply to this question, we may take a rapid survey of the data upon which he should rely.

1. The whole quantity of poisonous substance which has been swallowed is not absorbed, save in exceptional cases. A portion, usually the most considerable, is rejected by vomiting, or, after traversing the digestive canal, passes off by stool. When death takes place promptly, a portion may still be found in the canal. The remainder is absorbed, that is, it is carried into all the tissues. 2. The portion absorbed is not distributed uniformly through the various parts of the organism. Daily experience shows that with the same weights the liver always yields to analysis a much larger proportion of poison than all the other organs. 3. Elimination commences a short time after absorption, and the investigations which have been made up to the present time show that as regards certain of these poisons, arsenic, for example, it may be completed fifteen or twenty days after ingestion. It is therefore evident that the amount of poison remaining in the organs, where it has been carried by absorption, continues diminishing from the period of its ingestion, so that if life be sufficiently prolonged, the entire quantity of poison absorbed may have become eliminated prior to death. Thus, a month after the ingestion of an arsenical preparation the organs will contain no trace of arsenic. Chemical search will not discover it, but yet death has been none the less the result of the disturbance which it has caused. It is obvious that the presence of poisonous substance in the organism until the last moment is not necessary for the production of death. The blow was struck at an early period, diseased action has been developed under the influence of the poison, and death is the termination of such disease. 4. When the rejected matters are not handed over to the expert, and when the alimentary canal is completely empty, chemical research can only be directed to certain organs or portions of them. It is a rule for the first experts to retain a portion of the matters handed over to them for ulterior research. It is easy to understand, then, especially with the more or less considerable losses rendered unavoidable in so difficult an analysis, that the quantity found constitutes but a very small fraction of that which has been swallowed, or even of that which has been absorbed. 5. Finally, it is highly important to note that for no poison do we know the quantity sufficient to induce the accidents of poisoning. By observations, whose rarity retains their significance, we know, to some extent, the limit beyond which doses should, in general, be regarded as poisons; but this limit is incontestably very superior to the reality. Thus, we know that fifty centigrammes of arsenic will induce poisoning in all cases, save in very rare exceptions; but no one can decide, in a given instance, whether five, ten, twelve, or fifteen centigrammes will not suffice to cause death. The action of poisons is so variable, and we are so little acquainted with the causes which give rise to their variance, that we cannot, without risking to expose ourselves to serious error, determine what is the minimum dose of a poison sufficient in a particular case to give rise to death, or to the symptoms observed.

From the above data it results: 1. When the vomited matters and the stools have not been submitted to chemical analysis, it is impossible to ascertain even approximatively the quantity that has been swallowed; and consequently to declare whether the quantity ingested has been sufficient to cause death. The quantity absorbed, moreover, by reason of the unequal dissemination through the various organs, and the elimination which may have taken place, cannot be estimated by the amount found in certain organs or portions of organs. Such amount is but a minute fraction of the portion absorbed, which itself is but a fraction of the quantity swallowed. It may be sufficient to induce death, although the quantity ingested, or even that absorbed, may be more than sufficient. 2. If there have been neither vomiting nor stools, and if the digestive canal contain no poison, the quantity ingested has been all absorbed; and for the above reasons there is no means of judging by the quantity found of the amount which has been swallowed. 3. If the vomits and stools have been analyzed, or if (in their absence) the matters contained in the canal comprised the whole of the poison swallowed, except the portion absorbed, it is possible to declare approximatively the amount ingested. In such a case it is desired to establish whether the quantity ingested is sufficient to determine death. It is only when the quantity swallowed has been much greater than that which would be really sufficient for this purpose that the expert can reply affirmatively. Under other circumstances, by reason of our ignorance concerning the conditions which modify the action of poisons, the greatest reserve is peremptory. The reasons for doubt, dependent upon the actual state of science, should be stated; and this conclusion brought out that, in general, even when the quantity ingested has been really larger than that which is necessary to induce death, we are still not in a condition to affirm that it has induced it.

It is very desirable that this question of quantity should be reduced to exacter proportions, and that it should no longer contain the exaggerated importance which is now attributed to it. The symptoms and the lesions of tissue, combined with the detection of poison in the organs, are the most valuable elements for forming a decision whether poisoning has taken place, while the question of quantity, so far from bringing out the truth, will only conceal it in the great majority of cases. When a poison exists in organs or in matters which should not contain it, its quantity, save in some exceptional cases, cannot be insisted upon as a proof of poisoning. The great bulk of crimes of this kind would escape detection if such a proof were considered necessary. In order to exhaust this subject of quantity, and of the conclusions to be arrived at by experts respecting it, we may state two particular cases in which dosage is daily referred to. 1. Has the poison, which has been detected, resulted from the substance having been administered medicinally? An instant's reflection will suffice to explain that except in the case wherein the poison has been found in the matters of the alimentary canal, the vomit, or the stools, in quantities much larger than the highest therapeutical doses, the consideration of quantity will in nowise elucidate the problem. The portion absorbed, which, moreover, it is impossible to determine, will never be sufficiently considerable to allow of a sure deduction being made. 2. But it may be said, when we have to do with poisons which also exist in the animal economy in a normal state, is it not of service to have recourse to the consideration of quantity, in order to determine whether a poison discovered in the organs does not result rather from this normal combination than from a criminal introduction? When there exists in these organs a much larger quantity of poison than has been found in the normal condition, (and thus far experience has taught us very little on this matter,) we may be authorized to admit poisoning as probable, supposing that all other objections have been previously refuted. But when the amount discovered is not very considerable, (and, judging from the preceding considerations, this will be the usual case, inasmuch as it can be only a portion of what has been absorbed,) the embarrassment is just as great, and the question of quantity is of no significance.—(*Gazette des Hôpitaux*, No. 60, and *Druggist's Circular*, March, 1861.)

